# Clinical and Biochemical Phenotype of Adolescent Males with Gynecomastia

**DOI:** 10.4274/jcrpe.galenos.2019.2019.0027

**Published:** 2019-11-22

**Authors:** Miłosz Lorek, Dominika Tobolska-Lorek, Barbara Kalina-Faska, Aleksandra Januszek-Trzciakowska, Aneta Gawlik

**Affiliations:** 1 Medical University of Silesia Faculty of Medicine, Department of Pediatrics and Pediatric Endocrinology, Katowice, Poland

**Keywords:** Gynecomastia, puberty, estradiol, testosterone, ratio

## Abstract

**Objective:**

Gynecomastia is defined as a benign proliferation of male breast glandular tissue. Its prevalence during puberty varies between 50-60% and is also common in neonatal and elderly males. It develops mainly due to the disequilibrium between estrogen and androgen activity in breast tissue, where estradiol (E2) binds to estrogen receptors and stimulates ductal and glandular cells. The aim of this work was to investigate the relationship between sex hormone alterations and the natural history of gynecomastia.

**Methods:**

Participants in this study were young males referred to an outpatient clinic, between January 2011 and February 2016, with breast enlargement. Thyroid function, liver function, hormone concentrations and tumor markers were measured and anthropometric assessment was conducted.

**Results:**

Subjects comprised 93 males, aged 9 to 18 (mean±standard deviation age 13.8±2.6) years. In 63 of 93 (67.7%) the gynecomastia was confirmed and 28 were followed-up for a median period of three months. None of the boys showed any reduction in breast size during follow-up. There was no correlation between body mass index Z-score and breast size. Breast enlargement progressed in nine boys (32.1%). A positive correlation between estrogen to testosterone (E2/TTE) ratio and Tanner B stage (r=0.47; p=0.034) was observed.

**Conclusion:**

The E2/TTE ratio may be a helpful tool in diagnosing gynecomastia. Altered E2/TTE ratio might be responsible for a proportion of cases described previously as idiopathic. Additionally, weight loss does not imply reduction of breast size in boys. Nonetheless it should be the first step in the management of prolonged gynecomastia.

**What is already known on this topic?**

The imbalance between estrogen and androgen activity is considered to be the main cause of this gynecomastia. Nonetheless, studies that actually present data on the estrogen to testosterone (E2/TTE) ratio in gynecomastia patients are scarce.

**What this study adds?**

This study describes the relationship between alterations in sex hormones and the evolution of gynecomastia. Altered E2/TTE ratio might be responsible for part of cases described previously as idiopathic. This study highlights the importance of checking the E2/TTE ratio in gynecomastia patients.

## Introduction

Gynecomastia is defined as a benign, unilateral or bilateral proliferation of male breast glandular tissue. It is the most common breast alteration in males and has a trimodal age distribution, occurring in neonatal, pubertal, and elderly males. Gynecomastia is observed in 50% to 60% of boys during their puberty, usually bilaterally. It may be asymmetric in size (1,2). Physiologically gynecomastia has been reported to resolve within six months to two years after onset. Persistance indicates presence of a pathology which requires further evaluation (3,4,5,6).

The imbalance between estrogen and androgen activity is considered to be responsible for gynecomastia (7,8). Gynecomastia occurs in response to increased estrogen production and/or activity or because of decreased production and/or activity of testosterone (TTE) (9). Increased prevalence of gynecomastia raises the question of a factor which is associated with the pathophysiologic mechanism of gynecomastia.

A rapid increase in estradiol (E2), occurring before and delaying a similar increase in TTE, causes an elevated E2/TTE ratio at the onset of puberty. E2 binds to estrogen receptors in the breast tissue and stimulates ductal and glandular cell proliferation, leading to gynecomastia. Opposing this effect, TTE exerts a generalized inhibitory action on growth and differentiation, perhaps through a specific anti-estrogenic action (10).

Aromatase, which converts androstenedione and TTE to estrone and E2, respectively, is the most important factor in establishing equilibrium (11). Overexpression and increased activity of aromatase is a key factor in development of gynecomastia. This upregulation contributes to excessive local production of estrogen, decreased estrogen degradation and changes in the levels or activity of estrogen or androgen receptors (12,13).

Gynecomastia is strongly associated with obesity (14,15). Aromatization takes place in the adipose tissue and it is the main source of E2 in men. Subsequently, higher production and activity of aromatase are the key factors leading to gynecomastia in obese men (6,16). Furthermore, the increased body weight contributes to breast tissue proliferation by increased leptin level (12). Due to an increasing prevalence of adolescent obesity, it is essential to identify patients with gynecomastia among all boys presenting with breast enlargement.

Testicular tumors, as well as adrenal tumors, may secrete estrogen, causing disruption in the E2/TTE ratio (17,18). In addition, all forms of male hypogonadism lead to TTE deficiency, disrupting sex hormone homeostasis (4).

In addition, puberty is the period of the fastest linear growth in children, as remonstrated by peak height velocity (PHV) and at that time, insulin-like growth factor-1 (IGF-1) and growth hormone (GH) reach maximum levels. Both GH and IGF-1 are responsible for linear growth and also stimulate breast tissue proliferation through their respective receptors located in breast tissue (19). Occurrence of PHV and gynecomastia in a similar period of a young boy’s life may suggest that there is a relationship between them (20,21).

There are also other causes of gynecomastia that should not be disregarded, such as drug-induced gynecomastia, systemic illness and familial disorders. Common drug contributors include antipsychotics, antiretrovirals and prostate cancer therapies with long-term use (22,23). Gynecomastia is also a common sign of chronic liver disease and human immunodeficiency virus infection (24,25).

In some cases, the etiology remains uncertain and a pathomechanism responsible for gynecomastia cannot always easily be determined. A medical history of all young boys who present with gynecomastia should be carefully reviewed and each patient should be subjected to a thorough physical examination.

## Methods

All study subjects presented to the Upper Silesian Child Health Centre in Katowice. 93 male patients, ages ranged from 9 to 18 years with a mean±standard deviation (SD) age of 13.8±2.6 years at presentation, were referred to our endocrine outpatient clinic because of breast enlargement and were examined between January 2011 and February 2016. Of these, 11 were excluded due to steatomastia and 19 due to a reduction in breast size at the time of consultation. Sixty-three boys were diagnosed with gynecomastia and enrolled in the study group. Follow-up visits every 3-6 months were planned. Of these 63 subjects, two had a family history of gynecomastia and three had delayed puberty. There were also four cases of hyperprolactinemia and one patient had an additionally history of galactorrhoea concurrent with a normal prolactin (PRL) level. Thus the pathological gynecomastia group consisted of 11 patients and were compared to the rest, described as pubertal gynecomastia (n=52). None of the patients had a history of primary hypogonadism, drug-induced gynecomastia, a human chorionic gonadotropin (hCG)-secreting tumor or elevated aminotransferase concentrations.

The patients were divided into two groups with respect to Tanner breast development criteria. Thus, 42 of the 63 boys (66.7%) were classified as Tanner stage 2 (B2) of breast development and 21 boys (33.3%) as >B2.

### Clinical Phenotype

Anthropometric measurements included weight and height measurements. Body mass index (BMI) was calculated using the standard formula of weight (kg) divided by height (m) squared. Weight was measured using a scale with a precision of 100 g. Height was measured by a stadiometer sensitive to 0.1 cm. Height SD score (hSDS) was calculated from population standards for healthy children using the following formula: hSDS=child’s height-height for 50 pc/0.5 ∗ (height 50 pc-height 3 pc). Short stature was defined as hSDS below -2.0 SD.

Given a child’s age, sex, BMI, and the appropriate reference standard, the BMI Z-score was calculated using The Pediatric Z-score Calculator. The tool is available at the website of The Children’s Hospital of Philadelphia, Research Institute (http://stokes.chop.edu/web/zscore/) and can be used for subjects aged between two and 20 years. A BMI Z-score over +2.0 SD was classified as obesity, between +2.0 and +1.0 SD as overweight, between -1.0 and -2.0 as underweight and under -2.0 SD as significant weight deficiency (26). The boys’ sexual maturity stages were assessed using the Tanner scale (27).

### Biochemical Phenotype

E2, TTE, luteinizing hormone (LH), follicle-stimulating hormone (FSH) and PRL blood concentrations were measured using a chemiluminescent immunoassay by Immulite 2000 kit (Diagnostic Products Corp., Los Angeles, CA, USA). Serum free thyroxine (fT4) and thyroid stimulating hormone (TSH) concentrations were measured with a chemiluminescent immunometric assay (Immulite 2000 Free T4 Siemens Healthcare Diagnostics, Immulite 2000 Third Generation TSH, Diagnostic Products Corp., Los Angeles, CA, USA). Based on standardized E2 and TTE results (see the Statistical Analysis section below), the E2/TTE ratio was calculated. To exclude other causes of breast enlargement, such as cirrhosis and testicular tumors, alpha-fetoprotein (AFP), hCG, alanine transaminase (ALT) and aspartate transaminase (AST) were also measured, in accordance with International Federation of Clinical Chemistry (Beckman Coulter, USA).

### Statistical Analysis

All statistical analyses were performed using a Statistica 13 PL software (StatSoft Inc., Tulsa, Oklahoma, USA). A p value of <0.05 was considered significant. Shapiro-Wilk test was utilized to verify the normality of E2 and TTE distribution. In order to calculate the E2/TTE ratio, raw results were compared by using Standard Score. The analysis was stratified by gynecomastia status. The comparisons between two parametric values were made by using Student’s T-test or Mann-Whitney U test for non-parametric distributions. The correlation between quantitative values was analyzed by using Pearson’s correlation and Spearman’s rank correlation coefficient for ordinal variables. All results were reported as mean±SD.

### Ethics

All procedures in our study were performed in accordance with the ethical standards of the institutional and national research committee and with the 1964 Helsinki Declaration. Due to its retrospective design and non-experimental nature, a formal consent or a formal approval by a bioethics committee were not required.

## Results

### Clinical Results

The mean±SD age of the patients was 13.8±2.6 years at the time of the first consultation. At the initial assessment, seven (11.1%) boys were obese, 24 (38.1%) were overweight, 29 (46.0%) had a normal weight and three (4.8%) boys had weight deficiency. Mean±SD BMI was 22.9±4.3, mean±SD BMI Z-score was 0.83±1.0 and the mean±SD height SDS was 0.5±1.3. Among all 63 patients, only 28 (44.4%) turned up to scheduled visits in the endocrinology outpatient clinic and the longest follow-up lasted seven visits extending to 38 months. Fourteen (50%) boys were advised to lose weight and eight (28.6%) children followed the recommendation. Two of 14 (14.3%) of them achieved normal BMI Z-score, but reduction in size of breast during the observation period was not noted. There was no correlation between BMI Z-score and breast size (p>0.05). Breast size at Tanner B stage of 16/28 (57.1%) boys did not change. Breast enlargement progressed in 12/28 (42.9%) of the boys.

Gynecomastia was bilateral in 46/63 (73.0%) of the subjects. The median B Tanner stage at presentation was B2 (n=42; 67.0%). B3 stage was reported in 15 (23.8%) cases and six (9.5%) patients had B4 stage. Tanner stage 4 for pubic hair appeared most often (n=13, 20.6%), and mean±SD testicular volume was 12.2±5.5 mL when gynecomastia was observed for the first time.

The clinical characteristics of the patients with gynecomastia, divided into two groups (B2 or >B2), are shown in Table 1. There were no statistically signiﬁcant clinical differences between early and more advanced stage of the disease.

We were able to identify the cause of gynecomastia in 11 cases (17.4%) which were classified as the pathological gynecomastia group (n=11). In this group the mean±SD age was 14.9±3.0 years, mean±SD hSDS was 0.3±1.0 and mean±SD BMI Z-score was 0.6±0.8 at first presentation. These results did not differ statistically from the pubertal gynecomastia group.

### Hormonal Results

**Figure 1:**
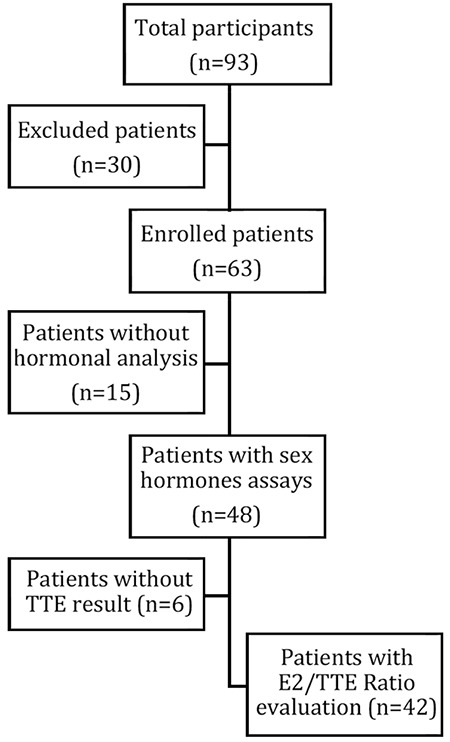
Flow chart of patients included in E2/TTE ratio evaluation E2/TTE: estrogen to testosterone

**Figure 2:**
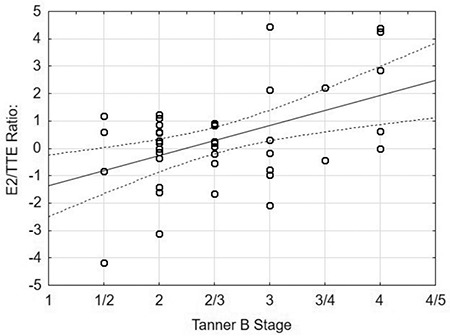
Correlation between E2/TTE ratio and the Tanner scale B stage E2/TTE: estrogen to testosterone

Hormonal results of all patients are presented in Table 2. E2 concentration was elevated in six (12.5%) boys. Of these, one was obese and two were overweight. There were five (10.9%) patients with TTE results below the reference interval and two of them were also overweight. None of the patients had elevated E2 and decreased TTE simultaneously Figure 1 displays a flow chart of patients included in E2/TTE ratio evaluation. A statistically significant positive correlation between E2/TTE ratio and Tanner B stage was found (r=0.47; p=0.034, see Figure 2). E2/TTE ratio did not correlate with BMI Z-score. The mean basal LH and FSH concentrations were in the pubertal ranges. TSH concentrations were elevated in six (12.8%) boys, although all of them had normal fT4 concentration. Among these, one boy was obese and four were overweight. Four (11.1%) boys had hyperprolactinemia (617.2; 604.6; 567.4 and 381.6 mIU/L) and one patient had a history of galactorrhoea concurrent with normal PRL level. ALT, AST, hCG and AFP of all patients were within normal ranges.

**Figure 3:**
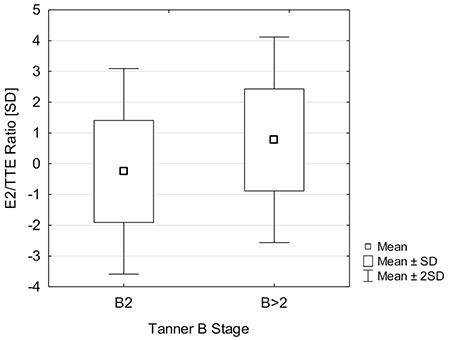
Comparison of E2/TTE ratio stratified by gynecomastia status; p=0.057 E2/TTE: estrogen to testosterone, SD: standard deviation

**Figure 4:**
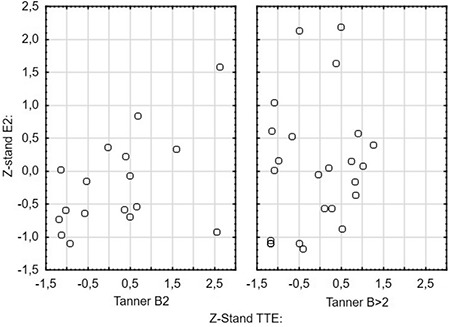
Scatter plot of standarised E2 and TTE values stratified by Tanner B stage TTE: testosterone, E2: estradiol

The comparison of B2 and >B2 groups revealed that patients with a breast stage >2 Tanner B tended to have higher E2/TTE ratios (0.8±1.8 versus -0.3±1.5; p=0.057; Figure 3, 4). There were no other differences between groups and all results are presented in Table 3.

Additionally, we investigated the clinical profile of six boys (14.3% out of 42 boys who had calculated E2/TTE ratio) whose E2/TTE ratio was over +1 SD (3.4±1.1). These boys were in the middle of puberty (14.7±2.1 years of age) with a BMI Z-score of 1.0±0.6 and a hSDS value of -0.1±1.5. All were at Tanner stage B3 (n=2) or B4 (n=4). They had TTE concentrations within the reference interval (242.3±97.7 ng/dL). Mean±SD E2 concentration among this group was 40.3±7.6 pg/mL and three boys had elevated E2 level (45.9±4.5 pg/mL) while three had normal E2 levels (34.2±4.9 pg/mL).

Finally, we analysed hormonal results in the pathological gynecomastia group, which were available for nine of 11 (81.8%) patients. Median (range) values for E2 were 23.8 (50.5) pmol/l and for TTE were 209.8 (825.4) ng/L ng/L. Mean E2/TTE ratio was 0.2±1.8. Due to the small number of subjects with hormonal results in this group, comparison with the pubertal gynecomastia group would be unreliable and was not undertaken.

## Discussion

Gynecomastia may be a cause of psychosocial discomfort, stress and worsening of self-image in a boy. It is important to understand these concerns in order to provide proper management. The suggested diagnostic approach and treatment strategies for gynecomastia consist of expert opinion, case series and follow-up observation, implying that the quality of evidence is not satisfactory and and that an unequivocal management appraoch regarding this problem is lacking.

This article reviews the validity of calculating E2/TTE ratio as a diagnostic aid in management of gynecomastia in young and adolescent boys. Increased E2/TTE ratio has been suggested as the main cause of gynecomastia (28,29,30,31). According to the literature, 25% of cases with gynecomastia are described as idiopathic (14,32). We have observed that no clear etiology for breast enlargement can be established in almost 65% of the patients, while sex hormone disturbances or other identifiable cause were present in 35% of the study subjects.

According to latest recommendations, each patient suspected of gynecomastia on physical examination without an identified cause, should undergo hormonal investigation including determination of blood LH, FSH, PRL, TTE, E2, beta-hCG and TSH (3,4). In our cohort only 2/3 of gynecomastia patients had undergone such an evaluation. Moreover, physicians should pay specific attention to “red flags” suggestive of non-physiologic gynecomastia (2,8,10). We did not observe rapid growth of breasts, breast skin changes, firm breast mass, testicular mass nor other signs of malignancy in this series. Only one boy was followed-up for longer than two years. In this case, we were able to diagnose persistent gynecomastia (>2 years), which is considered a “red flag” of pathological gynecomastia. One boy had a history of galactorrhea, which is also a cause for clinical concern, as nipple discharge may suggest a serious underlying pathological etiology for the of gynecomastia.

The average age of our patients at the first visit was 13.8±2.6 years and their level of pubic hair development which was at Tanner stage 3 were consistent with previous studies (33,34). On the other hand, about 40% of the study subjects were at more advanced Tanner stages for pubic hair and almost 35% had greater testicular volumes (P4G4) than is usually observed (P3G3) at this age (22).

The fact, that most of our patients did not have any abnormalities in basal hormone levels encouraged us to calculate the E2/TTE ratio, which was possible in 42 patients. We found that there was a weak correlation between the imbalance of estrogen and androgen, expressed as E2/TTE ratio, and breast size. This finding suggests that there may be a possibility of sex hormones disturbances in gynecomastia patients, despite E2 and TTE serum concentrations being with the appropriate reference ranges. Nonetheless, lack of differences between the B2 and >B2 groups for E2 and TTE concentrations and in their respective E2/TTE ratios suggests that caution should be exercised in drawing conclusions.

To the best of our knowledge, there are no specific guidelines on how to calculate E2/TTE ratio and there is no proposed cut off level for this parameter. In our study, the group of boys with higher (+1 SD) E2/TTE ratio values also showed higher E2 levels (40.3 *vs* 24.2 pg/mL) and more advanced breast tissue development. Nonetheless, we did not find any additional causes and features of gynecomastia in this subgroup. The imbalance in the E2/TTE ratio may explain why some adolescents with “normal” hormone levels develop gynecomastia. We propose that a cutoff point needs should be established for E2/TTE ratio following large, well-designed studies for use in clinical practice.

A rapid increase in obesity among children and adolescents results in a higher number of patients presenting with breast enlargement. Despite the fact that obesity causes pseudogynecomastia, that is a proliferation of adipose rather than glandular tissue, true gynecomastia is also associated with higher body weight. Rivera et al. indicated that there is a correlation between pubertal gynecomastia and higher BMI percentiles. Kulshreshtha et al (34) also reported that most of the patients (64%) with breast enlargement were obese as per Coles criteria. In our study, the subjects had higher BMI values than the general population according to Centers for Disease Control growth charts. However, we did not find a relationship between BMI Z-score and breast size. Despite the fact that eight overweight children (57.1%) succeeded in losing some weight, breast size was not reduced in any of them and weight changes did not affect sex hormone levels, as there was no correlation between BMI Z-score and E2/TTE ratio. This observation, that weight loss alone will not correct true glandular breast enlargement is consistent with that reported by other authors (17,36).

It should be also underlined that in the majority of our patients the breasts did not show an increase in size, while in a minority (19%) progression of breast size was evident. Adolescents with gynecomastia should be encouraged to lose weight, because it may complicate the surgical treatment of long-standing gynecomastia. Handschin et al (37) report that in adults with gynecomastia who are overweight, more severe surgical complications are observed and larger resections are needed.

Further studies should be performed in order to measure whether the same findings are applicable to the active fractions of sex hormones, that is free TTE and free E2 and studies should also take into account sex hormone binding globulin. Such studies might be helpful in providing greater guidance in differentiating true gynecomastia from lipomastia and pseudogynecomastia, especially in obese boys.

### Study Limitations

We are aware of the limitations of the retrospective design of our study. As the study was limited to a single center, the number of study subjects was also restricted. The smallness of the sample may have led to overlooking the actual correlations. Moreover, the study was conducted in a single region of Poland, Silesia. Despite these limitations, our study design provides some valuable estimates, as each patient underwent the same process of diagnosis and the hormonal results were established in the same laboratory.

## Conclusions

In conclusion, our results show that the E2/TTE ratio may be a helpful tool in diagnosing gynecomastia. We speculate that an altered E2/TTE ratio might be responsible for a portion of the cases previously described as idiopathic. Additionally, weight loss does not imply reduction of breast size in boys. Nonetheless, it should be the first step before further treatment of prolonged gynecomastia.

## Figures and Tables

**Table 1:**
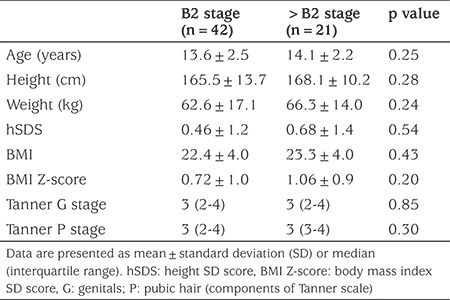
Clinical characteristics of adolescent boys with gynecomastia stratified by Tanner B stage

**Table 2:**
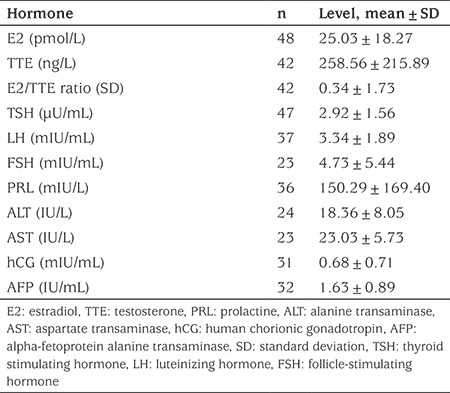
Hormone levels in all boys with pubertal gynecomastia

**Table 3:**
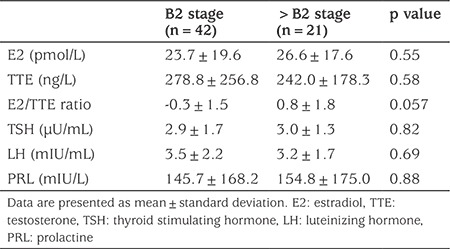
Biochemical characteristics of adolescent boys with gynecomastia stratified by Tanner B stage

## References

[1] Johnson RE, Murad MH (2009). Gynecomastia: Pathophysiology, Evaluation, and Management. Mayo Clin Proc.

[2] Dickson G (2012). Gynecomastia. Am Fam Physician.

[3] Nordt CA, DiVasta AD (2008). Gynecomastia in adolescents. Curr Opin Pediatr.

[4] Sansone A, Romanelli F, Sansone M, Lenzi A, Di Luigi L (2017). Gynecomastia and hormones. Endocrine.

[5] Akgül S, Derman O, Kanbur N (2017). Pubertal gynecomastia: years of progress - the Hacettepe experience. Int J Adolesc Med Health.

[6] Simoni M, Huhtaniemi I, eds. Endocrinology of the Testis and Male Reproduction. 2017:1-21.

[7] Carlson HE (2011). Approach to the patient with gynecomastia. J Clin Endocrinol Metab.

[8] Braunstein GD (1993). Gynecomastia. N Engl J Med.

[9] Durmaz E, Ozmert EN, Erkekoglu P, Giray B, Derman O, Hincal F, Yurdakök K (2010). Plasma phthalate levels in pubertal gynecomastia. Pediatrics.

[10] Jameson JL, De Groot LJ. Endocrinology: Adult and Pediatric, 2-Volume Set, 7th Edition. 2016.

[11] Eren E, Edgunlu T, Korkmaz HA, Cakir E, Demir K, Cetin ES, Celik SK (2014). Genetic variants of estrogen beta and leptin receptors may cause gynecomastia in adolescent. Gene.

[12] Dundar B, Dundar N, Erci T, Bober E, Büyükgebiz A (2005). Leptin levels in boys with pubertal gynecomastia. J Pediatr Endocrinol Metab.

[13] Bembo SA, Carlson HE (2004). Gynecomastia: Its features, and when and how to treat it. Cleve Clin J Med.

[14] Rahmani S, Turton P, Shaaban A, Dall B (2011). Overview of gynecomastia in the modern era and the leeds gynaecomastia investigation algorithm. Breast J.

[15] Daniels IR, Layer GT (2001). Gynaecomastia. Eur J Surg.

[16] Rokutanda N, Iwasaki T, Odawara H, Nagaoka R, Miyazaki W, Takeshita A, Koibuchi Y, Horiguchi J, Shimokawa N, Iino Y, Morishita Y, Koibuchi N (2008). Augmentation of estrogen receptor-mediated transcription by steroid and xenobiotic receptor. Endocrine.

[17] Rosen H, Webb ML, DiVasta AD, Greene AK, Weldon CB, Kozakewich H, Perez-Atayde AR, Labow BI (2010). Adolescent Gynecomastia: not only an obesity issue. Ann Plast Surg.

[18] Yazici M, Sahin M, Bolu E, Gok DE, Taslipinar A, Tapan S, Torun D, Uckaya G, Kutlu M (2010). Evaluation of breast enlargement in young males and factors associated with gynecomastia and pseudogynecomastia. Ir J Med Sci.

[19] Sansone A, Romanelli F, Sansone M, Lenzi A, Di Luigi L (2017). Gynecomastia and hormones. Endocrine.

[20] Mieritz M, Sorensen K, Aksglaede L, Mouritsen A, Hagen CP, Hilsted L, Andersson AM, Juul A (2014). Elevated serum IGF-I, but unaltered sex steroid levels, in healthy boys with pubertal gynaecomastia. Clin Endocrin (Oxf).

[21] Juul A, Holm K, Kastrup KW, Pedersen SA, Michaelsen KF, Scheike T, Rasmussen S, Müller J, Skakkebaek NE (1997). Free insulin-like growth factor I serum levels in 1430 healthy children and adults, and its diagnostic value in patients suspected of growth hormone deficiency. J Clin Endocrinol Metab.

[22] Limony Y, Friger M, Hochberg Z (2013). Pubertal Gynecomastia Coincides with Peak Height Velocity. J Clin Res Pediatr Endocrinol.

[23] Roke Y, van Harten PN, Boot AM, Buitelaar JK (2009). Antipsychotic medication in children and adolescents: a descriptive review of the effects on prolactin level and associated side effects. J Child Adolesc Psychopharmacol.

[24] Bowman JD, Kim H, Bustamante JJ (2012). Drug-induced gynecomastia. Pharmacotherapy.

[25] Biglia A, Blanco JL, Martínez E, Domingo P, Casamitjana R, Sambeat M, Milinkovic A, Garcia M, Laguno M, Leon A, Larrousse M, Lonca M, Mallolas J, Gatell JM (2004). Gynecomastia among HIV-infected patients is associated with hypogonadism: a case-control study. Clin Infect Dis.

[26] Braunstein GD (2004). Editorial comment: unraveling the cause of HIV- related gynecomastia. AIDS Read.

[27] Such K, Gawlik A, Dejner A, Wasniewska M, Zachurzok A, Antosz A, Gawlik T, Malecka-Tendera E (2016). Evaluation of Subclinical Hypothyroidism in Children and Adolescents: A Single-Center Study. Int J Endocrinol.

[28] Marshall WA, Tanner JM (1970). Variations in the Pattern of Pubertal Changes in Boys. Arch Dis Child.

[29] Nuttall FQ, Warrier RS, Gannon MC (2015). Gynecomastia and drugs: A critical evaluation of the literature. Eur J Clin Pharmacol.

[30] Ankarberg-Lindgren C, Norjavaara E (2008). Twenty-four hours secretion pattern of serum estradiol in healthy prepubertal and pubertal boys as determined by a validated ultra-sensitive extraction RIA. BMC Endocr Disord.

[31] Thiruchelvam P, Walker JN, Rose K, Lewis J, Al-Mufti R (2016). Gynaecomastia. BMJ.

[32] Meikle AW (2004). The interrelationships between thyroid dysfunction and hypogonadism in men and boys. Thyroid.

[33] Al Alwan I, Al Azkawi H, Badri M, Tamim H, Al Dubayee M, Tamimi W (2013). Hormonal, anthropometric and lipid factors associated with idiopathic pubertal gynecomastia. Ann Saudi Med.

[34] Kulshreshtha B, Arpita A, Rajesh PT, Sameek B, Dutta D, Neera S, Mohd M (2017). Adolescent gynecomastia is associated with a high incidence of obesity, dysglycemia, and family background of diabetes mellitus. Indian J Endocrinol Metab.

[35] Rivera N, Eisenstein E, Cardoso CB (2009). The relation between pubertal gynecomastia and body mass index in a sample of adolescents attended at the Outpatient Health Unit of a University Hospital. Arq Bras Endocrinol Metabol.

[36] Nuzzi LC, Cerrato FE, Erikson CR, Webb ML, Rosen H, Walsh EM, DiVasta AD, Greene AK, Labow BI (2013). Psychosocial impact of Adolescent gynecomastia. Plast Reconstr Surg.

[37] Handschin AE, Bietry D, Hüsler R, Banic A, Constantinescu M (2008). Surgical management of gynecomastia -- a 10-year analysis. World J Surg.

